# Glucose-1,6-bisphosphate: A new gatekeeper of cerebral mitochondrial pyruvate uptake

**DOI:** 10.1016/j.molmet.2024.102018

**Published:** 2024-08-24

**Authors:** Motahareh Solina Safari, Priska Woerl, Carolin Garmsiri, Dido Weber, Marcel Kwiatkowski, Madlen Hotze, Louisa Kuenkel, Luisa Lang, Matthias Erlacher, Ellen Gelpi, Johannes A. Hainfellner, Gottfried Baier, Gabriele Baier-Bitterlich, Stephanie zur Nedden

**Affiliations:** 1 CCB-Biocenter, Institute of Neurobiochemistry, Medical University of Innsbruck, 6020 Innsbruck, Austria; 2 Department of Biochemistry, Institute of Bioanalytic & Intermediary Metabolism, University of Innsbruck, 6020 Innsbruck, Austria; 3 CCB-Biocenter, Institute of Genomics and RNomics, Medical University of Innsbruck, 6020 Innsbruck, Austria; 4 Department of Neurology, Division of Neuropathology and Neurochemistry, Medical University of Vienna, 1090 Vienna, Austria; 5 Institute for Cell Genetics, Medical University of Innsbruck, 6020 Innsbruck, Austria

**Keywords:** Energy metabolism, Phosphoglucomutase 2 like 1, Glucose-1,6-bisphosphate, Mitochondrial pyruvate carrier, Ischemia, Protein kinase N1

## Abstract

**Objective:**

Glucose-1,6-bisphosphate (G-1,6-BP), a byproduct of glycolysis that is synthesized by phosphoglucomutase 2 like 1 (PGM2L1), is particularly abundant in neurons. G-1,6-BP is sensitive to the glycolytic flux, due to its dependence on 1,3-bisphosphoglycerate as phosphate donor, and the energy state, due to its degradation by inosine monophosphate-activated phosphomannomutase 1. Since the exact role of this metabolite remains unclear, our aim was to elucidate the specific function of G-1,6-BP in the brain.

**Methods:**

The effect of PGM2L1 on neuronal post-ischemic viability was assessed by siRNA-mediated knockdown of PGM2L1 in primary mouse neurons. Acute mouse brain slices were used to correlate the reduction in G-1,6-BP upon ischemia to changes in carbon metabolism by ^13^C_6_-glucose tracing. A drug affinity responsive target stability assay was used to test if G-1,6-BP interacts with the mitochondrial pyruvate carrier (MPC) subunits in mouse brain protein extracts. Human embryonic kidney cells expressing a MPC bioluminescence resonance energy transfer sensor were used to analyze how PGM2L1 overexpression affects MPC activity. The effect of G-1,6-BP on mitochondrial pyruvate uptake and oxygen consumption rates was analyzed in isolated mouse brain mitochondria. PGM2L1 and a predicted upstream kinase were overexpressed in a human neuroblastoma cell line and G-1,6-BP levels were measured.

**Results:**

We found that G-1,6-BP in mouse brain slices was quickly degraded upon ischemia and reperfusion. Knockdown of PGM2L1 in mouse neurons reduced post-ischemic viability, indicating that PGM2L1 plays a neuroprotective role. The reduction in G-1,6-BP upon ischemia was not accompanied by alterations in glycolytic rates but we did see a reduced ^13^C_6_-glucose incorporation into citrate, suggesting a potential role in mitochondrial pyruvate uptake or metabolism. Indeed, G-1,6-BP interacted with both MPC subunits and overexpression of PGM2L1 increased MPC activity. G-1,6-BP, at concentrations found in the brain, enhanced mitochondrial pyruvate uptake and pyruvate-induced oxygen consumption rates. Overexpression of a predicted upstream kinase inhibited PGM2L1 activity, showing that besides metabolism, also signaling pathways can regulate G-1,6-BP levels.

**Conclusions:**

We provide evidence that G-1,6-BP positively regulates mitochondrial pyruvate uptake and post-ischemic neuronal viability. These compelling data reveal a novel mechanism by which neurons can couple glycolysis-derived pyruvate to the tricarboxylic acid cycle. This process is sensitive to the glycolytic flux, the cell's energetic state, and upstream signaling cascades, offering many regulatory means to fine-tune this critical metabolic step.

## Highlights

• Glucose-1,6-bisphosphate (G-1,6-BP) is specifically elevated in the brain and quickly degraded upon energetic stress.
• The G-1,6-BP synthase Phosphoglucomutase 2 like 1 (PGM2L1) is important for post-ischemic neuronal survival.
• G-1,6-BP enhances mitochondrial pyruvate uptake and oxygen consumption rates.
• PGM2L1 activity and G-1,6-BP levels can be inhibited by Protein Kinase N1.

## 1 Introduction

The mammalian brain, one of the organs with the highest energy demands, mainly depends on glucose as its energy source [1]. Neurons can rely on glycolysis to supply the tricarboxylic acid (TCA) cycle [2], and pyruvate, due to its position at the crossroads of these two processes, occupies a central metabolic role. However, the mechanisms regulating the precise coupling of pyruvate to the TCA cycle in the brain under normal and pathological conditions remain to be elucidated.

An often-ignored byproduct of glycolysis is glucose-1,6-bisphosphate (G-1,6-BP), a metabolite synthesized by phosphoglucomutase 2 like 1 (PGM2L1) [3] and specifically elevated in the brain [4]. To form G-1,6-BP, PGM2L1 uses the glycolytic intermediate 1,3-bisphosphoglycerate as phosphate donor and glucose-1 or 6-phosphate as a phosphate acceptor [3,5,6]. While G-1,6-BP is a well-known cofactor for phosphoglucomutases, the cerebral concentrations of G-1,6-BP are higher than those needed to stimulate phosphoglucomutases [4]. Furthermore, G-1,6-BP levels in the brain are sensitive to the cellular energy state, as a rise in the adenosine triphosphate (ATP) degradation metabolite inosine monophosphate (IMP), results in the activation of the G-1,6-BP-phosphatase phosphomannomutase 1 (PMM1) [[7], [8], [9]]. Accordingly brain G-1,6-BP levels fall quickly in response to ischemia [4], an effect that is ameliorated in PMM1 knockout mice [9]. Therefore, G-1,6-BP acts as a sensor for both glycolytic flux and the energetic state of brain tissue.

It has been shown that several key steps of glycolysis can be regulated by G-1,6-BP, including the inhibition of hexokinase and the activation of phosphofructokinase and pyruvate kinase [[10], [11], [12], [13]]. However, these effects are more sensitively achieved by other metabolites and to the best of our knowledge, most evidence is based on enzymatic assays and correlative studies. Furthermore, the regional distribution of PGM2L1 in the brain coincides with that of G-1,6-BP and PMM1 [14], but it does not correlate with the distribution of phosphoglucomutases [15] or other enzymes involved in carbohydrate metabolism, nor with the rate of glucose consumption [16]. Since the regulatory effect of G-1,6-BP could not be conclusively linked to carbohydrate metabolism enzymes, it has been suggested that G-1,6-BP likely has other functions in the brain [15]. Theories range from G-1,6-BP being an alternative phosphate donor to ATP [6] to G-1,6-BP serving as an energy reserve for glucose-6-phosphate [17]. The importance of G-1,6-BP for normal cerebral function is underscored by the fact that impaired G-1,6-BP production, due to mutations in PGM2L1 in humans, is associated with a neurodevelopmental disorder. This condition is not caused by a glycosylation defect and somewhat resembles the lack of creatine kinase, suggesting that G-1,6-BP has unidentified functions in brain energy metabolism [18].

Therefore, the goal of this study was to investigate if G-1,6-BP regulates cerebral carbon metabolism. By relating the decrease in G-1,6-BP upon ischemia to changes in carbon metabolism fluxes we found a correlative reduction in ^13^C_6_-glucose incorporation into citrate. We show that G-1,6-BP enhances mitochondrial pyruvate uptake and pyruvate-induced oxygen consumption rates by activating the mitochondrial pyruvate carrier. We further show that PGM2L1 is important for post-ischemic neuronal cell survival and identify upstream kinases involved in the regulation of PGM2L1 activity. These exciting findings describe a novel regulatory mechanism of mitochondrial pyruvate uptake in the brain and establish future avenues for targeting post-ischemic neuronal survival.

## 2 Materials and methods

### 2.1 Mice

For all experiments adult (>1 month old) or one to two day old C57BL/6N mice were used. Male and female animals were used equally throughout this study. Animals were kept under standard housing conditions with a 12 h light/dark cycle. Animals younger than P12 were sacrificed by decapitation and animals older than P12 were sacrificed by cervical dislocation.

### 2.2 Acute brain slices

**Figure 1:**
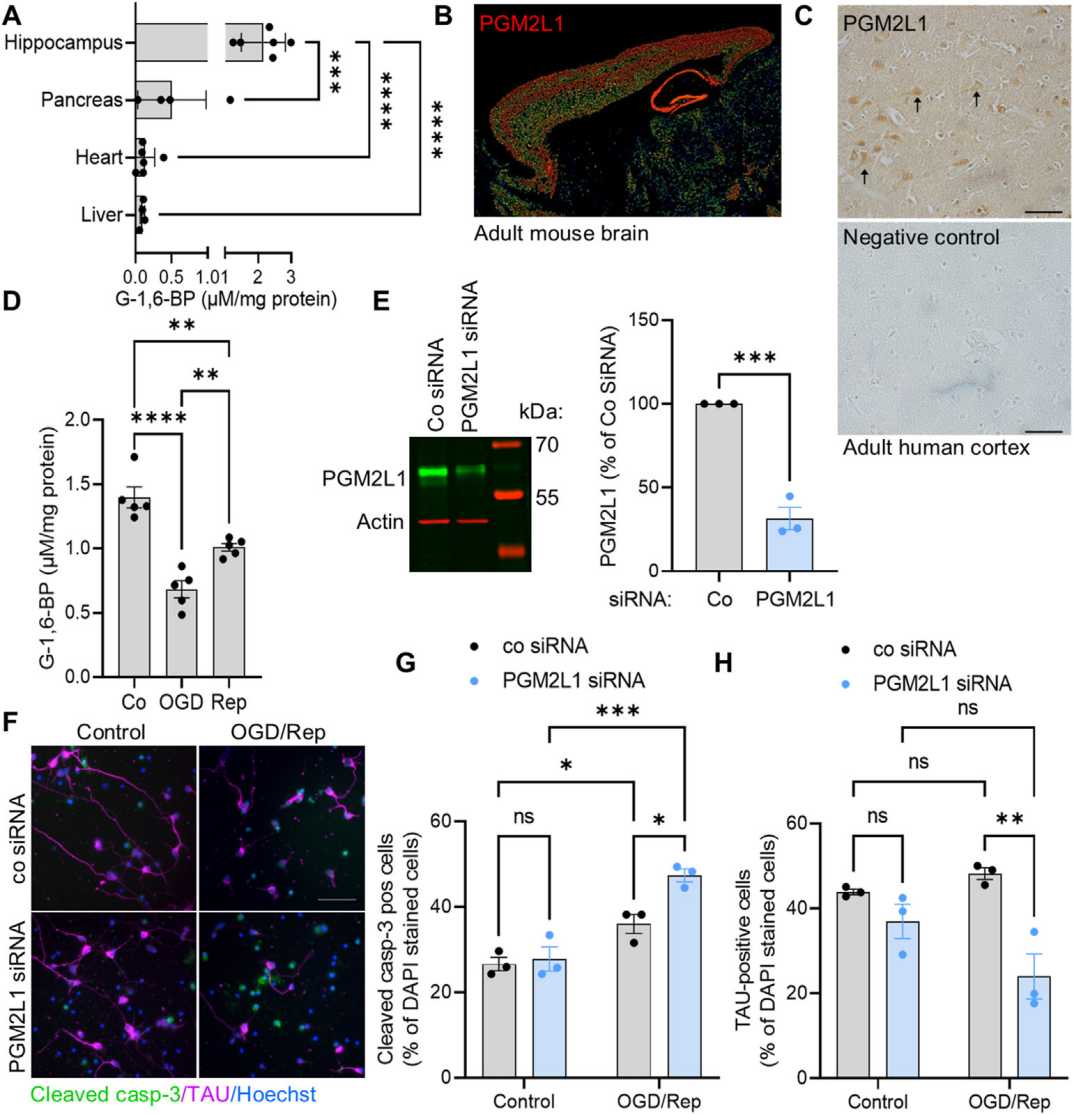
**PGM2L1 is important for post-ischemic neuronal survival.** (**A**) G-1,6-BP levels were measured in organs of adult mice (one way ANOVA with Holm-Šídák's multiple comparisons test, (∗∗∗) P < 0.001, (∗∗∗∗) P < 0.0001)). (**B**) Expression of PGM2L1 in adult mouse brain. Image credit: Allen Institute for Brain Science (Allen Brain atlas Pgm2l1 - RP_040825_02_D01 – sagittal, http://mouse.brain-map.org/experiment/show/545100). (**C**) Human prefrontal cortical sections were stained for PGM2L1 (see also Supplemental Fig. 1), which was mainly seen in pyramidal neurons, identified by their triangular shape (arrowheads). (**D**) Hippocampal mouse brain slices were exposed to control conditions, 7 min OGD or 7 min OGD and 2 h Rep and G-1,6-BP was measured (one way ANOVA with Holm-Šídák's multiple comparisons test, (∗∗) P < 0.01, (∗∗∗∗) P < 0.0001). (**E**) Cortical mouse neurons were transfected with non-targeting siRNA (Co siRNA) or PGM2L1 siRNA and knockdown was confirmed after 48 h by western blotting (unpaired t-test (∗∗∗) P < 0.001). (**F**) Transfected neurons were exposed to 3 h OGD and 1 h Rep and stained for Hoechst, cleaved caspase-3 (casp-3, green) and the neuronal marker TAU (magenta). Image is representative of 3 separate experiments. (**G**) The percentage of cleaved casp-3 positive cells was calculated (two way ANOVA: Treatment (∗∗∗) P < 0.001, siRNA (∗) P < 0.05, Interaction (∗) P < 0.05, Holm-Šídák's multiple comparisons test (∗) P < 0.05, (∗∗∗) P < 0.001, (ns) not significant). (**H**) The percentage of TAU-positive cells was calculated (two way ANOVA: Treatment P > 0.05, siRNA (∗∗) P < 0.01, Interaction (∗) P < 0.05, Holm-Šídák's multiple comparisons test (∗∗) P < 0.01, (ns) not significant). All scale bars refer to 50 μm. All data are presented as scatter blot with mean ± S.E.M.

Sagittal brain slices (400 μm), composed of hippocampus and overlying neocortex (except for Figure 1D where the cortex was removed), were prepared from adult mice as described previously [19], using a Leica Vibratome (VT1200S). Slices were allowed to recover for >2 h in continuously circulating, standard artificial (a) cerebrospinal fluid (CSF) at 34 °C, oxygenated with 95% O_2_/5% CO_2_. The composition of the standard aCSF solution included the following: NaCl 124 mM, KCl 3 mM, CaCl_2_ 2 mM, NaHCO_3_ 26 mM, NaH_2_PO_4_ 1.25 mM, d-glucose 10 mM, and MgSO_4_ 1 mM, pH 7.4. Oxygen glucose deprivation (OGD) was achieved by transferring slices into glucose-free aCSF containing an equimolar concentration of sucrose (10 mM) and pre-equilibrated (>1 h) with 95% N_2_/5% CO_2_. OGD was induced for 7 min, in order to achieve anoxic depolarization (which occurred after 5.6 ± 1.2 min (mean ± standard deviation)) and still allow for metabolic recovery. After OGD slices were transferred back into standard aCSF for indicated reperfusion (Rep) time points. For each condition and n-number two slices were used per animal.

### 2.3 Metabolite extraction for glucose-1,6-bisphosphate measurement

**Brain slices:** Mouse brain slices were lysed in 160 μl 5% perchloric acid (PCA, Sigma Aldrich, 244252) by homogenization with a handheld motor-driven pellet-pistille (Kimble, Sigma Aldrich). Extracts were centrifuged (10,000 g, 3 min, 4 °C) and 150 μl of the supernatant were neutralized with 19 μl 3M K_2_CO_3_, kept on ice for 5 min with an open lid and centrifuged again (10,000 g, 3 min, 4 °C). The neutralized supernatant was stored at −80 °C until analysis. The pellet was resuspended in 160 μl 1M NaOH and analyzed for the protein content (Pierce, BCA Protein assay, Thermo Fisher Scientific).

**SH-SY5Y cells:** 48 h after transfection, human SH-SY5Y cells (see Section 2.16) were harvested in ice cold PBS, centrifuged (1350 rpm, 5 min) and the pellet was lysed in PCA as described above.

**Tissues:** Mouse organs were removed in the following order: pancreas, lateral liver lobe, heart apex, immediately snap frozen in liquid N_2_ and stored at −80 °C until lysis (wet weight (wwt) liver: 60–100 mg; pancreas: 15–25 mg; heart: 35–65 mg). Isolated hippocampi were lysed immediately. PCA was added at a ratio of 160 μl/15 mg wwt, neutralized as described above and centrifuged (15,000 g, 7 min, 4 °C). Samples with pH < 7 were excluded and outliers were identified in GraphPad Prism 9, with the ROUT method (Q = 10 %).

**Mitochondria:** Isolated mitochondria (see Section 2.9) from mouse brain were kept for 7 min or 15 min at 30 °C in mitochondrial respiration buffer (see Section 2.13). Mitochondria were centrifuged (14,000 g, 5 min, 4 °C), washed with ice cold measurement buffer and centrifuged again. Metabolites were extracted by sonicating mitochondria in 80% acetonitrile.

### 2.4 Measurement of glucose-1,6-bisphosphate (G-1,6-BP)

G-1,6-BP was determined spectrophotometrically by stimulating the activity of muscle phosphoglucomutase and glucose-6-phosphate dehydrogenase as described previously [18]. The assay was downscaled to a 96 well plate format. Briefly, 2–2.5 μl of the PCA or mitochondrial acetonitrile extracts were diluted into an end volume of 10 μl double distilled H_2_O in a 96 well plate. The assay was started by adding 90 μl assay buffer (end-concentration: TRIS pH 7.1 50 mM, EGTA 0.1 mM, MgCl_2_ 5 mM, NADP^+^ 0.5 mM, glucose-1-phosphate 0.5 mM, glucose-6-phosphate dehydrogenase (Sigma–Aldrich, G6378-100UN) 1.75 U/ml, phosphoglucomutase (Sigma–Aldrich, P33997-200UN; 0.03 U/ml)). NADPH production was monitored after 35 min at 34 °C at a wavelength of 340 nm. The sample concentrations of G-1,6-BP were calculated from a standard curve (0–2 μM G-1,6-BP, Sigma Aldrich, G6893 and 49225) and related to mg protein or wet weight.

### 2.5 Preparation and transfection of cortical neurons

Cortical neurons were prepared from one to two day old mouse pups [20,21]. After decapitation and dissection both cortices were cut into small pieces in dissecting solution (HBSS (Sigma Aldrich, H9269), gentamycin (Sigma Aldrich, G1264, 20 μg/ml), MgCl_2_ 9 mM). The tissue was transferred into papain-HBSS solution (HBSS 1 ml, papain 2 mg (Worthington Biochemical Corporation, LS003119), DNAse 0.1 mg/ml (Sigma Aldrich, Roche 11284932001), MgCl_2_ 9 mM), incubated for 10 min at 37 °C and inverted 3 times. Papain activity was stopped by addition of 2 ml HBSS, supplemented with BSA (10 mg/ml), trypsin inhibitor (Gibco, Thermo Fisher Scientific, 17075029, 1 mg/ml) and MgCl_2_ (9 mM). The tissue was triturated with pasteur pipette tips of decreasing size and after centrifugation (2000 rpm, 5 min, room temperature) 1 × 10^7^ cells were transfected (Lonza, VPG-1001) with control non-targeting siRNA or PGM2L1 siRNA (Dharmacon, end-concentration 300 nM) using program G-013 (Amaxa Nucleofector II device). Cells were seeded on poly-l-ornithine (Sigma Aldrich, P4957)-coated coverslips and kept in Neurobasal Plus (Gibco, A3582901) supplemented with 1× B-27 (Gibco, 17504044) and 1% penicillin/streptomycin/glutamine (Sigma Aldrich, P4333). Cells were washed with glucose-free Neurobasal-A medium (Gibco, A2477501) and OGD was induced by addition of glucose-free supplemented Neurobasal-A medium (1× B27 and 1% penicillin/streptomycin/glutamine), which has been equilibrated in 95% N_2_/5%CO_2_ overnight in a modular incubator chamber (Billups-Rothenberg, MIC-101). Cells were returned to the anoxic atmosphere and kept for 3 h. The medium of control cells was exchanged for fresh, supplemented Neurobasal Plus medium. After 3 h the medium of all cells was exchanged with the preconditioned medium for 1 h reperfusion.

### 2.6 Immunofluorescence staining

Immunocytochemistry was performed as described previously [22]. Cells were fixed (4% PFA, 30 min), permeabilized (0.3% Triton X-100 in PBS, 30 min, room temperature), blocked (1 h, 10 % normal goat serum, 2% BSA in PBS) and primary antibodies (section 2.17) were added over night at 4 °C in antibody solution (5 % normal goat serum, 1 % BSA, 0.1 % Triton X-100, in PBS). After washing in PBS-T (0.05 % Tween) secondary antibodies (goat-anti rabbit Alexa-488 A11070, and goat anti-mouse Alexa-555 A21425, Molecular Probes, Life technologies, 1:1000), as well as Hoechst (8 μM, Molecular Probes, Life technologies), were added for 2–4 h at room temperature in antibody solution. Coverslips were washed in PBS-T and embedded in Mowiol (Sigma Aldrich). Images were taken with a widefield (Axio, Zeiss, Oberkochen, Germany) microscope.

### 2.7 ^13^C_6_-glucose tracing

After indicated treatments mouse brain slices were transferred into a 2 ml Eppendorf tube with oxygenated aCSF where glucose was substituted with 10 mM ^13^C_6_-glucose for 10 min. Slices were homogenized with a motor-driven pellet-pistille (Kimble, Sigma Aldrich) in 160 μl 100% methanol (kept at −20 °C) and the extract was centrifuged (16,000 g, 3 min, 4 °C). The supernatant was transferred into a fresh tube and dried in a SpeedVac (Savant, SC110A) and kept at −80 °C until analysis. The pellet was used to determine the wet weight.

### 2.8 Mass spectrometric analysis of ^13^C label enrichment

Dried methanol extracts were reconstituted in 200 μl of HPLC-grade water, and metabolic label enrichment was analysed using ion chromatography (IC, Dionex ICS-6000, Thermo Fisher Scientific) coupled to a quadrupole orbitrap mass spectrometer (MS, Exploris 480, Thermo Fisher Scientific). 4 μl of each sample was injected and separated using an IonPac AS11-HC column (2 × 250 mm, 4 μm, Thermo Scientific) equipped with an IonPac AG11-HC guard column, as described previously [23]. Briefly, the analytes were separated by a potassium hydroxide (KOH) gradient generated by an eluent generator and a flow rate of 380 μl/min using the following gradient: 0–3 mM KOH in 3 min, 3–10 mM KOH in 2 min, 10–30 mM KOH in 15 min, 30–50 mM KOH in 7 min, and 50–85 mM KOH in 2 min. For MS coupling, a make-up flow (methanol, 2 mM acetic acid) was introduced via a T-piece at a flow rate of 60 μl/min. The quadrupole orbitrap was equipped with a heated electrospray ion (HESI) source and the following source parameters were used for MS analysis: source temperature: 350 °C, sheath gas: 50 [a.u], auxiliary gas: 10 [a.u], auxiliary gas temperature: 350 °C, spray voltage: 2,500 V, ion transfer capillary temperature: 325 °C. MS analyses were performed in negative ion mode using RF-Lens of 50% for ion transmission. Full scan MS spectra were recorded over a *m*/*z* scan range of 50–750 *m*/*z*, a resolution of 60,000 fwhm at *m*/*z* 200, a maximum injection time of 50 ms, and an AGC target of 1 × 10ˆ6. Quantification of metabolite label enrichment was performed using targeted single ion monitoring (SIM) mode with the following parameters: resolution: 120,000 fwhm at *m*/*z* 200, maximum injection time: 246 ms, AGC target: 1 × 10ˆ5. The scan width and retention time window used to quantify the metabolite isotopologue species are listed in Table 1. Metabolite isotopologues were quantified with TraceFinder (version 5.0, Thermo Fisher Scientific). The Genesis algorithm was used for peak detection and integration with the following parameters: peak detection strategy: highest peak; peak threshold type: area; threshold: 1; smoothing: 3; S/N threshold: 3; tailing factor: 3. If necessary, peaks were adjusted manually. Peak areas were corrected for natural isotope abundance using PiCor: resolution correction for R = 120 k, *m*/*z* calibration for reference *m*/*z* = 200 (https://github.com/MolecularBioinformatics/PICor) [24]. Further data analysis and visualization was performed in R (version 4.3.1) and RStudio (version 2024.04.1 + 748).

**Table 1:** List of polar metabolites, charge, single ion monitoring (SIM) window and retention times used to quantify ^13^C label enrichment by IC-SIM-MS.

metabolite	charge [z]	SIM window [m/z]	t start [min]	t stop [min]
Hexose	[M−H]^−1^	177.056–187.056	1.5	3.5
Lactate	[M−H]^−1^	87.524–93.524	6	8
Pyruvate	[M−H]^−1^	85.509–91.509	7	9
Glucose-1-Phosphate	[M−H]^−1^	257.022–267.022	10	12.5
Succinate	[M−H]^−1^	115.019–123.019	12	14.5
Malate	[M−H]^−1^	131.014–139.014	12.5	15
Glucose-6-Phosphate, Fructose-6-Phosphate^a^	[M−H]^−1^	257.002–267.002	14.2	17.5
α-Ketoglutarate	[M−H]^−1^	143.014–152.014	14.5	17
Fumarate	[M−H]^−1^	113.004–121.004	15	18
Aconitate	[M−H]^−1^	171.009–181.009	25	29
Phospho(enol)pyruvate	[M−H]^−1^	165.475–171.475	25.5	29
Citrate, Isocitrate^a^	[M−H]^−1^	189.020–199.0120	23.5	27
Fructose-1,6-Bisphosphate, Glucose-1,6-Bisphosphate^a^	[M−H]^−1^	336.989–346.989	27	32

a Both isobaric metabolites were measured in the same SIM window, as they exhibited baseline separation in IC.

### 2.9 Isolation of mouse brain mitochondria

Mouse brain mitochondria were extracted with the mitochondrial isolation kit for tissue (Abcam, ab110168) with following adaptations: One hemisphere consisting of cortex and hippocampus was lysed in 2 ml isolation buffer. After centrifugation (1000 g, 10 min, 4 °C), the supernatant was transferred to 2 tubes and washed with 1 ml isolation buffer each. After centrifugation (12,000 g, 15 min, 4 °C), the pellet was resuspended in 200 μl 12 % Percoll (in isolation buffer) and layered onto 1 ml 24 % Percoll (in isolation buffer). This step was included to further purify mitochondria from synaptosomes and myelin [25]. The tubes were centrifuged (18,000 g, 18 min, 4 °C, brake and acceleration 4) and the top 700 μl were transferred to a new tube and washed with 1.2 ml isolation buffer. After centrifugation (18,000 g, 5 min, 4 °C), 1.5 ml supernatant were removed and another 1.5 ml isolation buffer were added and centrifuged again (14,000 g, 5 min, 4 °C). The pooled pellets were used for further experiments, as described in each section.

### 2.10 Protein extraction and western blotting

Protein extracts of mitochondria and cells were prepared in Tris Lysis buffer (Tris pH 8.5 50 mM, NP-40 1%, EDTA 5 mM, sodium pyrophosphate 5 mM, sodium fluoride 5 mM, NaCl 50 mM, activated sodium orthovanadate 5 mM, aprotinin 30 μg/ml, leupeptin 30 μg/ml) and analyzed by western blotting as described previously, using the LiCor Odyssey Imager [26].

### 2.11 Drug affinity responsive target stability (DARTS) assay

The DARTS assay is often used for small molecule target identification and is based on the principle that proteins become less susceptible to proteases when they bind to small molecules/metabolites. We performed the DARTS assay following a previously published protocol [27]. One hippocampus was lysed in 400 μl M-PER (Thermo Fisher Scientific, 78503) supplemented with a protease inhibitor cocktail (Sigma Aldrich, Roche, 11836153001). The lysate was centrifuged (18,000 g, 15 min, 4 °C) and 360 μl supernatant was mixed with 40 μl 10× TNC buffer (10× Tris–HCl 0.5 M pH 8.0, NaCl 0.5 M, CaCl_2_ 0.1 M). The lysate was further diluted with 400 μl 1× TNC buffer, which then gave a protein concentration of 2–5 mg/ml. The lysate was split into 50 μl aliquots, which were either treated with vehicle (double distilled H_2_O) or G-1,6-BP at indicated concentrations. For complex formation lysates were incubated for 60 min on ice, followed by 15 min at room temperature. Thereafter pronase (Sigma Aldrich, Roche, 10165921001) or vehicle (double distilled H_2_O) was added at indicated concentrations (mg pronase/mg protein lysate). Lysates were incubated for 30 min at 25 °C. The reaction was stopped by adding Laemmli buffer and incubating the lysates at 70 °C for 10 min. For lysates with low or high protease inhibitor cocktail treatment either vehicle (double distilled H_2_O) or an additional protease inhibitor cocktail (Sigma Aldrich, Roche 11697498001) was added before treatment with G-1,6-BP. All other steps were performed as described above without the addition of pronase.

### 2.12 ^14^C-pyruvate uptake in isolated mouse brain mitochondria

For measurement of mitochondrial ^14^C-pyruvate uptake the protocol described in [28] with following adjustments was used. The mitochondrial pellet of section 2.9 was resuspended in 480 μl pyruvate transport buffer (PTB) pH 7.4 (PTB7.4: sucrose 250 mM, KCl 80 mM, MgCl_2_ 5 mM, MOPS 10 mM, 0.1% BSA, pH 7.4) yielding a concentration of ∼2 mg/ml crude mitochondria. Per time point and treatment 52 μl of this suspension was mixed with 156 μl PTB pH 6.8 (PTB6.8: sucrose 250 mM, KCl 80 mM, MgCl_2_ 5 mM, MOPS 10 mM, 0.1% BSA, pH 6.8) supplemented with 50 μM ^14^C_3_-pyruvate (Hartmann Analytic GmbH, ARC 0220) and either vehicle (double distilled H_2_O) or 100 μM G-1,6-BP. All reactions were stopped at indicated time points by addition of 10 μM UK-5099 (Medchemexpress, HY-15475) and mitochondria were re-isolated by centrifugation (16.000 g, 1 min, 4 °C). The pellets were washed with 500 μl PTB7.4 containing 50 mM cold pyruvate and 10 μM UK-5099, and centrifuged again (16.000 g, 1 min, 4 °C). The pellets were resuspended in 5 ml scintillation cocktail and analyzed by scintillation counting. For each experiment a positive control (1 μl of PTB6.8 with 50 μM ^14^C_3_-pryuvate) and a blank control (PTB7.4) was analyzed. The blank was subtracted from all readings and the values were expressed as % of the positive control. Each sample was related to its control value (0 min) and used for analysis.

### 2.13 Measurement of oxygen consumption rates (OCR) of isolated mouse brain mitochondria

OCR of isolated mouse brain mitochondria were measured with the Extracellular Oxygen Consumption Assay Kit (Abcam, ab197243) as per manufacturer's instructions. The mitochondrial pellet of section 2.9 was resuspended in 520 μl OCR measurement buffer (sucrose 250 mM, KCl 15 mM, EGTA 1 mM, MgCl_2_ 5 mM, K_2_HPO_4_ 30 mM in double distilled H_2_O; pH 7.4) resulting in a protein concentration of ∼1.8 mg/ml. Substrate mix (pyruvate 5 mM, ADP 4 mM, malate 1 mM), 10 μl fluorescent probe and OCR measurement buffer (to bring the volume to 150 μl) were added to each well in triplicates and the plate was kept at 30 °C in the dark. Blank wells were filled with 200 μl OCR measurement buffer. 100 μM G-1,6-BP or vehicle (double distilled H_2_O) was added and the reaction was started by addition of 50 μl cold mitochondria. Each well was quickly layered with 2 drops of mineral oil and the plate was read in a plate reader over the course of 45 min (BMG Labtech CLARIOstar, at 30 °C, excitation 380 ± 15 nm, emission 650 ± 20 nm). The blank wells were subtracted from all readings and the slope was calculated for each treatment and used for analysis.

### 2.14 Determination of pyruvate dehydrogenase activity

The pyruvate dehydrogenase positive control of the pyruvate dehydrogenase activity assay was treated with vehicle (H_2_O) or 100 μM G-1,6-BP and the activity was analyzed as per manufacturer's instructions (Sigma–Aldrich, MAK183).

### 2.15 Bioluminescence resonance energy transfer (BRET) assay and transfection of HEK cells

A genetically encoded biosensor based on bioluminescence resonance energy transfer (BRET) was used to monitor the activity of the mitochondrial pyruvate carrier (MPC). BRET is based on the fact that the energy from a luciferase reaction can be used for the excitation of a fluorescent protein when the protein is in close proximity to the luciferase enzyme. The technology involves the fusion of donor (luciferase) and acceptor (fluorescent) molecules to proteins of interest [29]. We kindly received human embryonic kidney (HEK) cells expressing the RESPYR construct (for REporter Sensitive to PYRuvate, MPC1-mVenus and MPC2-RLuc8) [29] from Dr. Kaushik Bhattacharya and Dr. Jean-Claude Martinou. Cells were kept in DMEM (FCS 10%, glutamine 2% and penicillin/streptomycin 1%, 95% humidity, 37 °C). Human PGM2L1 was purchased from GeneCopoeia (Plasmid EX-T4519-M02) and nucleofection (Lonza Nucleofection reagent V (VCA-1003)) was performed with 5 μg control plasmid (empty vector M02) or 5 μg PGM2L1 plasmid using program Q-001 (Amaxa Nucleofector II device) and 4 × 10^6^ cells/transfection. Cells were seeded in triplicates/quadruplicates in white clear bottom 96 well plates (2 × 10^4^ cells/well) or 60 mm dishes and used for the BRET assay or protein extraction 48 h after transfection. Immediately before starting the BRET assay, cells were washed and the medium was exchanged for PBS-C/M (supplemented with CaCl_2_ 1 mM and MgCl_2_ 0.5 mM). 5 μM coelenterazine H (Thermo Fisher Scientific, C6780) was added and cells were incubated for 5–10 min in the dark at 37 °C, thereafter the baseline reading was started (460 ± 20 nm (RLuc8 filter) and 535 ± 20 nm (Venus filter), 1 s measurement time). Substances were added manually, the plate was returned and the reading continued for indicated time points. Results were expressed as ratio (535 nm/460 nm) normalized to the mean of the baseline. For transfected HEK cells the PBS-readings were subtracted from the respective pyruvate readings. Care was taken to quickly record the BRET ratio after changing the medium to PBS-C/M, to avoid substantial degradation of G-1,6-BP. For permeabilization of the cells saponin (25 μg/ml, Calbiochem, 558255) was added together with coelenterazine H for 15 min and cells were treated as described above.

### 2.16 Culture and transfection of human SH-SY5Y cells

SH-SY5Y cells (ATCC, Manassas, VA) were cultured in DMEM (FCS 10%, penicillin/streptomycin 1%). Human (*h*) PKN1 was purchased from GeneCopoeia (Rockville, MD, USA) and subcloned into the mammalian expression vector pTO_HA_StrepIII_c_GW_FRT_EF1alpha as described in [30]. Per transfection 1 × 10^7^ cells were mixed with 10 μg of indicated plasmids: pTOO-HA (empty vector for PKN1), *h*PKN1-HA tagged, M02 (empty vector for PGM2L1), M02-untagged PGM2L1 and nucleofected (VPG-1001, Lonza) using program G-004 (Amaxa Nucleofector II device). Cells were analyzed for their G-1,6-BP levels after 48 h.

### 2.17 Antibodies

All antibodies used throughout the study are shown below with their catalogue numbers, applications and dilutions.

**Table:** Antibodies

Target	Company (catalogue number)	Application	Dilution
PGM2L1	Proteintech (#13942-1-AP)	W-Blot IF IHC	1:1000 1:250 1:100
MPC1	Cell Signaling (#14462)	W-Blot	1:1000
MPC2	Proteintech (#20049-1-AP)	W-Blot	1:1000
GAPDH	Abcam (#ab9484)	W-Blot	1:1000
Actin	Merck (#MAB1501)	W-Blot	1:5000
CoxIV	Proteintech (#11242-1-AP)	W-Blot	1:1000
ATPB	Proteintech (#66600-1-Ig)	W-Blot	1:1000
Cleaved Caspase 3	Cell Signaling (#94530)	IF	1:300
TAU	Cell signaling (#4019)	IF	1:500
VDAC1	Biolegend (#820702)	IF	1:100
HA	Cell signaling (#3724)	W-Blot	1:1000

### 2.18 Immunohistochemistry of human brain tissue

Sections of formalin-fixed paraffin-embedded human frontal cortex tissue (5 μm thickness) were generously provided by Dr. Ellen Gelpi, Dr. Gabor Kovacs and Dr. Johannes Hainfellner (Medical University of Vienna). Sections were deparaffinized in xylene twice for 10 min, followed by dehydration in ethanol (100%–95%–70%–50%, 5 min each). Antigen retrieval was performed in citrate buffer (citric acid 10 mM, pH 6.0, 95 °C, 15 min) and endogenous peroxidase activity was blocked (3% hydrogen peroxide, 10 min). The sections were blocked, permeabilized and primary antibodies were added as described in section 2.6. After washing in PBS, the slides were incubated with a secondary antibody conjugated to horseradish peroxidase (goat Anti-rabbit Immunoglobulins/HRP, Dako, #P0448) for 2 h. The signal was intensified using the VECTASTATIN Elite ABC kit (VectorLabs, PK-6100), and ImmPACT DAB substrate solution (VectroLabs, #SK-4105) was applied to visualize the staining. Staining intensity was monitored until the desired level was achieved, and the reaction was stopped by rinsing the slides in distilled water. After dehydration and mounting, the stained tissue sections were examined under a brightfield microscope, and images were captured for further analysis. Control sections were treated in the same way without adding primary antibodies.

### 2.19 Statistical analysis

All statistical analyses were performed in GraphPad Prism 9. All data are presented as individual n-values with or as mean ± S.E.M. For brain slice experiments n-values refer to two slices/condition from separate animals. n-values from mitochondrial preparations, DARTs assays, BRET assays and cell culture experiments, refer to different animals or transfections, except for the ^14^C_3_-pyruvate uptake where two hemispheres from one animal were used as separate n-values, from a total of 3–6 animals. For comparison of two independent groups, a two-tailed t-test was used. For comparison of three or more groups, a one way ANOVA with Holm-Šídák's multiple comparisons test was used. For comparison of two variables of two groups a two way ANOVA with Holm-Šídák's multiple comparisons test was used. Multiple t-tests with a false discovery rate of 1% employing the two-stage set-up were used to compare citrate isotopologues. P-values smaller than 0.05 were considered as statistically significant.

### 2.20 Ethics statement

**Animal work:** All procedures were performed in compliance with relevant laws and institutional guidelines. Animal work has been conducted in Austria. Animal work has been conducted in accordance with Directive 2010/63EU and ETS 123. According to article 3 of Directive 2010/63/EU sacrificing of animals solely for the use of their organs and tissues is not classified as a procedure. All staff members involved have been adequately trained and every effort was made to minimize the number of animals used.

**Human brain staining:** Ethical approval for the use of human postmortem brain tissue for research purposes was obtained from the Medical University of Vienna (EK1454/2018).

## 3 Results

### 3.1 G-1,6-BP is sensitive to the energetic state of the tissue and important for survival of post-ischemic neurons

PGM2L1 expression and G-1,6-BP levels have been shown to be particularly elevated in the brain [4,5]. By comparing G-1,6-BP concentrations in various mouse tissues, we were able to confirm that hippocampal G-1,6-BP levels were higher than those found in heart, pancreas and liver (Figure 1A). Within the mouse brain PGM2L1 is predominantly expressed in glutamatergic neurons (Dataset: Allen Institute for Brain Science (2020). Allen Cell Types Database - Mouse Whole Cortex and Hippocampus. Available from celltypes.brain-map.org/rnaseq. RRID:SCR_019013) [31], with highest expression levels in the entorhinal cortex and the pyramidal cell layer of the hippocampus [15] (Figure 1B) [[32], [33], [34], [35]]. In human brain, PGM2L1 is also mainly found in glutamatergic neurons (https://www.proteinatlas.org/ENSG00000165434-PGM2L1) [36], and staining of human frontal cortical tissue showed that PGM2L1 is strongly expressed in pyramidal neurons (Figure 1C, Supplemental Fig. 1).

G-1,6-BP levels have been shown to be sensitive to the energetic state of the tissue, since accumulation of the ATP degradation metabolite IMP results in activation of PMM1, which then degrades G-1,6-BP to glucose-6-phosphate [9]. We confirmed this in an *in vitro* ischemia-reperfusion model using acute mouse hippocampal brain slices subjected to oxygen glucose deprivation (OGD) and reperfusion (Rep). G-1,6-BP levels decreased during OGD and did not completely recover during Rep (Figure 1D). To understand if PGM2L1 plays a detrimental or crucial role in cell survival following energetic stress we knocked down PGM2L1 in cortical mouse neurons (Figure 1E) and analyzed post-ischemic cell viability. OGD/Rep resulted in a significant increase in cleaved caspase (casp)-3-positive cells, which was further enhanced upon knockdown of PGM2L1 (Figure 1 F, G). In addition, knockdown of PGM2L1 resulted in a reduction of TAU-positive neurons after OGD/Rep (Figure 1F,H), further showing that PGM2L1 is important for post-ischemic neuronal cell viability. Accordingly, PGM2L1 and its products, G-1,6-BP and potentially other hexose or pentose bisphosphates, exert neuroprotective actions during energetic stress. To elucidate a potential role of G-1,6-BP in carbohydrate metabolism, we utilized its sensitivity to ischemia/reperfusion to examine if its drop correlated with changes in glycolytic flux and the tricarboxylic acid (TCA) cycle in mouse brain slices.

### 3.2 The energetic stress-induced reduction in G-1,6-BP is accompanied by a reduced incorporation of ^13^C_6_-glucose into citrate

**Figure 2:**
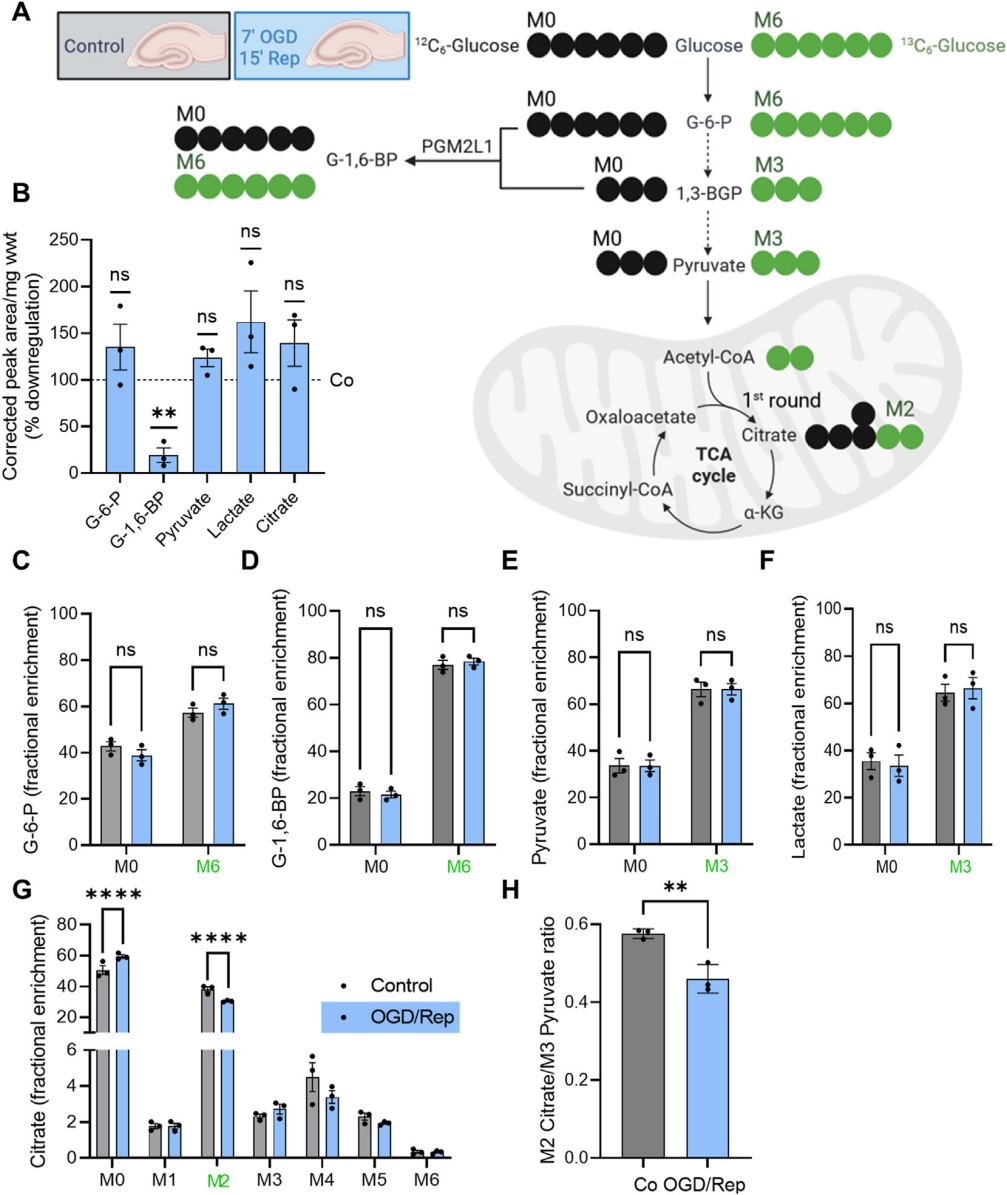
**Reduction in G-1,6-BP levels is accompanied by reduced incorporation of**^**13**^**C**_**6**_**-glucose into M2 citrate.** (**A**) Mouse hippocampal/neocortical brain slices were exposed to control conditions or 7 min OGD and 15 min Rep. Thereafter slices were incubated in aCSF containing 10 mM ^13^C_6_-glucose (M6) for 10 min. Metabolites were analyzed for the fractional enrichment of ^13^C into glycolytic and TCA metabolites (Supplemental Table 1). M0 refers to “normal” ^12^C-metabolites. Image was prepared in BioRender. (**B**) The abundance (corrected peak area/mg wwt) of glucose-6-phosphate (G-6-P), pyruvate, lactate or citrate was not affected by OGD/Rep. There was a significant reduction in G-1,6-BP. Data is presented as % downregulation compared to control conditions (one sample t-test compared to hypothetical value of 100%, (∗∗) P < 0.01, (ns) not significant). There was no difference (not significant (ns)) in ^13^C incorporation into (**C**) G-6-P, (**D**) G-1,6-BP, (**E**) pyruvate or (**F**) lactate. (**G**) There was a significant reduction in fractional enrichment of M2 citrate (multiple t-test with a false discovery rate (Q) of 1% employing the two-stage set-up, (∗∗∗∗) P < 0.0001). (**H**) The reduction in M2 citrate was not due to upstream differences in glycolysis as analyzed by the M2 citrate/M3 pyruvate ratio (unpaired t-test, (∗∗) P < 0.01). All data are presented as scatter blot with mean ± S.E.M.

G-1,6-BP has been suggested to regulate various enzymes involved in glycolysis, such as hexokinase, phosphofructokinase and pyruvate kinase [[10], [11], [12], [13]], however the relevance of these effects in the cell or *in vivo* is not clear. We therefore analyzed whether the ischemia-induced decrease in mouse hippocampal G-1,6-BP content was paralleled by changes in the flux or content of metabolites involved in glycolysis and the TCA cycle using ^13^C_6_-glucose tracing (Figure 2A, Supplemental Table 1 for all metabolites). We first analyzed how metabolite levels were affected by ischemia/reperfusion. OGD/Rep resulted in a reduction in G-1,6-BP levels while other glycolytic and TCA metabolites such as glucose-6-phosphate (G-6-P), pyruvate, lactate or citrate levels were not affected (Figure 2B). The incorporation rate of ^13^C_6_-glucose into G-6-P (Figure 2C), G-1,6-BP (Figure 2D), pyruvate (Figure 2E) or lactate (Figure 2F) was not altered by OGD/Rep, suggesting that G-1,6-BP levels do not correlate with the glycolytic flux. The only correlative change with respect to the reduction in G-1,6-BP levels was a reduced flux of ^13^C_6_-glucose into mitochondrial citrate (Figure 2G), specifically M2 citrate, which corresponds to the first round of the TCA cycle (Figure 2A). The M2 citrate/M3 pyruvate ratio was significantly smaller after OGD/Rep, indicating that mechanisms other than the glycolytic flux must be responsible for the reduced incorporation of M3 pyruvate into citrate after ischemia (Figure 2H). Therefore, we next tested if G-1,6-BP regulates mitochondrial pyruvate uptake or metabolism.

### 3.3 G-1,6-BP affects MPC activity and stimulates mitochondrial pyruvate uptake

**Figure 3:**
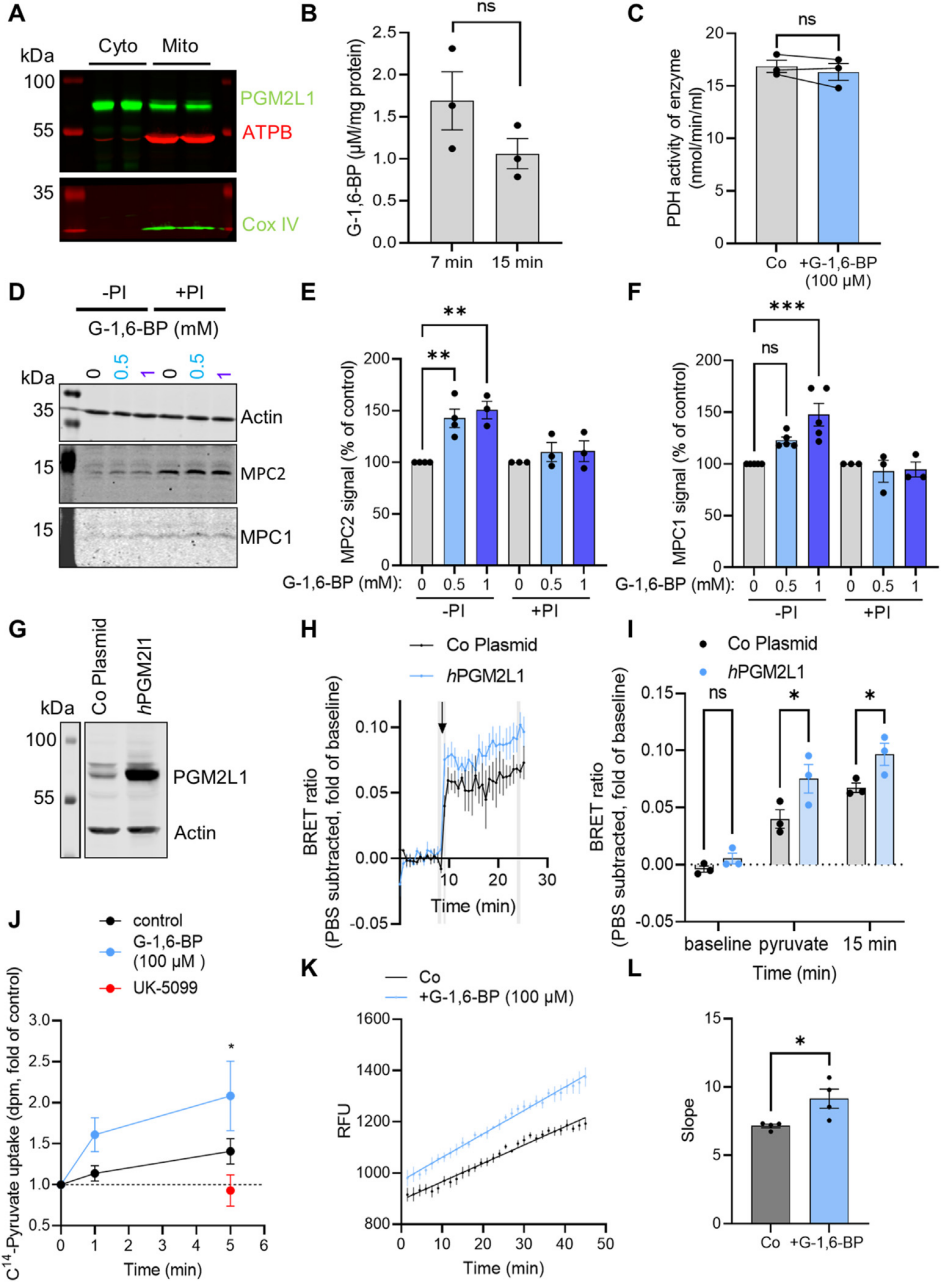
**G-1,6-BP regulates mitochondrial pyruvate uptake.** (**A**) Protein from mouse brain mitochondria was isolated and probed for mitochondrial markers (ATPB and Cox IV) and PGM2L1, which was localized to both, the cytosolic (Cyto) and mitochondrial fraction (Mito). Blot is representative of 3 separate experiments. (**B**) The mitochondrial G-1,6-BP content remained stable over a 15 min incubation period (unpaired t-test, P > 0.05). (**C**) Addition of G-1,6-BP (100 μM) to pyruvate dehydrogenase (PDH) did not alter its activity (paired t-test, P > 0.05). (**D**) A DARTS assay was performed with mouse hippocampal protein extracts incubated with low (-PI) or high protease inhibitor cocktail (+PI) after addition of vehicle (double distilled H_2_O, 0 mM) or 0.5 mM or 1 mM G-1,6-BP. G-1,6-BP (0.5 mM and 1 mM) protected (**E**) MPC2 from degradation by endogenous proteases (one way ANOVA with Holm-Šídák's multiple comparisons test: (∗∗) P < 0.01), while (**F**) MPC1 was only protected by 1 mM G-1,6-BP (one way ANOVA with Holm-Šídák's multiple comparisons test: (∗∗∗) P < 0.001, (ns) not significant). The protective effects were lost upon incubation with high PI (+PI) cocktail. (**G**) Control plasmids (empty vector, Co Plasmid) or human (*h*) PGM2L1 were overexpressed in human HEK cells expressing MPC1-mVenus (V)/MPC2-RLuc8 (R). Blot is representative of 3 separate experiments. (**H**) Transfected HEK MPC1V/MPC2R were treated with pyruvate (5 mM, arrowhead) or PBS and the BRET ratio was monitored. Values are expressed as fold of baseline with the PBS values subtracted (see Supplemental Figs. 3E and F for raw values). Areas marked in grey were used for analysis (N = 3). (**I**) The mean of 2 values (baseline and 15 min after addition of pyruvate) and the first value after addition of pyruvate of the areas marked in grey in H were compared (two way ANOVA Plasmid (∗∗∗∗) P < 0.0001, treatment (∗∗∗) P < 0.001, interaction P > 0.05, Holm-Šídák's multiple comparisons test (∗) P < 0.05). (**J**) Brain mitochondria were isolated and ^14^C_3_-pyruvate uptake was measured in the presence of vehicle (double distilled H_2_O, control, N = 8), 100 μM G-1,6-BP (N = 6) or 10 μM UK-5099 (N = 4). All values were related to each respective time point 0 (0 min, which was immediately stopped by the addition of 10 μM UK-5099). Pyruvate uptake with 0 and 100 μM G-1,6-BP was compared (two way ANOVA, Time (∗∗∗) P < 0.001, Treatment (∗∗) P < 0.01, Interaction P > 0.05, Holm-Šídák's multiple comparisons test: (∗) P < 0.5). (**K**) Mouse brain mitochondria were isolated and analyzed for their oxygen consumption rates (OCR). Mitochondria were stimulated with pyruvate (5 mM), ADP (4 mM) and malate (1 mM) and the effect of 100 μM G-1,6-BP on pyruvate-mediated OCR was analyzed. Raw relative fluorescence units (RFU) values are shown. (**L**) The slopes of each curve (from 1 to 45 min, shown in K) of the response was analyzed (unpaired t-test, (∗) P < 0.05). All data are presented as scatter blot with mean ± S.E.M., except for H, J and K were only mean ± S.E.M is shown.

We first analyzed the subcellular localization of PGM2L1. Western blots of mouse brain mitochondrial and cytosolic fractions (Figure 3A) as well as immunohistochemical analysis of mouse brain sections (Supplementary Fig. 2) revealed that PGM2L1 was found in the cytosol. However, we observed a partial colocalisation with a mitochondrial marker (Supplementary Fig. 2), and PGM2L1 was also detected in the mitochondrial fraction of mouse brains (Figure 3A), suggesting a potential association of PGM2L1 with this organelle. The mitochondrial localization of PGM2L1 is further supported by data from the Human Protein Atlas (https://www.proteinatlas.org/ENSG00000165434-PGM2L1) [36]. Similar to liver mitochondria [37], we were able to measure G-1,6-BP in isolated brain mitochondria, suggesting that this metabolite is taken up by or diffuses into mitochondria, and remains stable over a short incubation period (Figure 3B). This further supports a potential role of G-1,6-BP in mitochondrial metabolism.

G-1,6-BP had no effect on pyruvate dehydrogenase activity (Figure 3C). We therefore analyzed if G-1,6-BP interacts with the mitochondrial pyruvate carrier (MPC), a heterodimeric transporter composed of MPC1 and MPC2 [38,39], by employing a drug affinity responsive target stability assay (DARTS). This assay can be used to study protein-molecule interactions and is based on the principle that proteins become less susceptible to proteases when bound to small molecules/metabolites [40]. We found that G-1,6-BP protects MPC2 from degradation by endogenous proteases and at higher concentrations also MPC1 (Figure 3D–F). Addition of an exogenous protease (Supplemental Fig. 3A) further confirmed that result, showing that MPC2 (Supplemental Figs. 3A and B) was protected from proteasomal degradation to a greater extent than MPC1 (Supplemental Figs. 3A and C). Therefore, G-1,6-BP interacts with the MPC subunits, particularly MPC2. We next tested if PGM2L1 regulates MPC activity by using human HEK cells expressing the RESPYR sensor composed of MPC1-mVenus and MPC2-RLuc8 [29]. This sensor measures BRET activity in response to a conformational change in MPC1 and MPC2 subunits which can be induced either upon pyruvate transport or upon inhibition of the MPC activity using small molecule inhibitors, such as UK-5099 [29,41] (Supplemental Fig. 3D). HEK cells overexpressing human (*h*) PGM2L1 (Figure 3G) showed a significantly stronger BRET response to addition of pyruvate than HEK cells transfected with a control plasmid (Figure 3H,I, Supplemental Figure 3 E,F). Since PGM2L1 can also synthesize other bisphosphates, such as mannose-1,6-bisphosphate and ribose-1,5-biosphosphate [5] we wanted to confirm that G-1,6-BP regulates MPC activity. Exogenous addition of G-1,6-BP to permeabilized HEK cells resulted in stabilization and stronger MPC activity (Supplemental Figure 3 G,H).

To further assess whether G-1,6-BP increases or inhibits MPC-mediated pyruvate transport we used a mitochondrial ^14^C_3_-pyruvate uptake assay [28]. Addition of G-1,6-BP to isolated mouse brain mitochondria, at concentrations found in the brain (100 μM) [9], enhanced mitochondrial pyruvate uptake (Figure 3J). To test if this was also translated into enhanced mitochondrial oxygen consumption rates (OCR) we incubated isolated mouse brain mitochondria with pyruvate, malate and adenosine diphosphate (ADP) (Figure 3K) and found that G-1,6-BP was able to enhance pyruvate-induced OCR (Figure 3K,L). Therefore, our results show that G-1,6-BP is a regulator of mitochondrial pyruvate uptake and pyruvate-induced OCR by directly stimulating MPC activity.

### 3.4 Regulation of PGM2L1 activity

**Figure 4:**
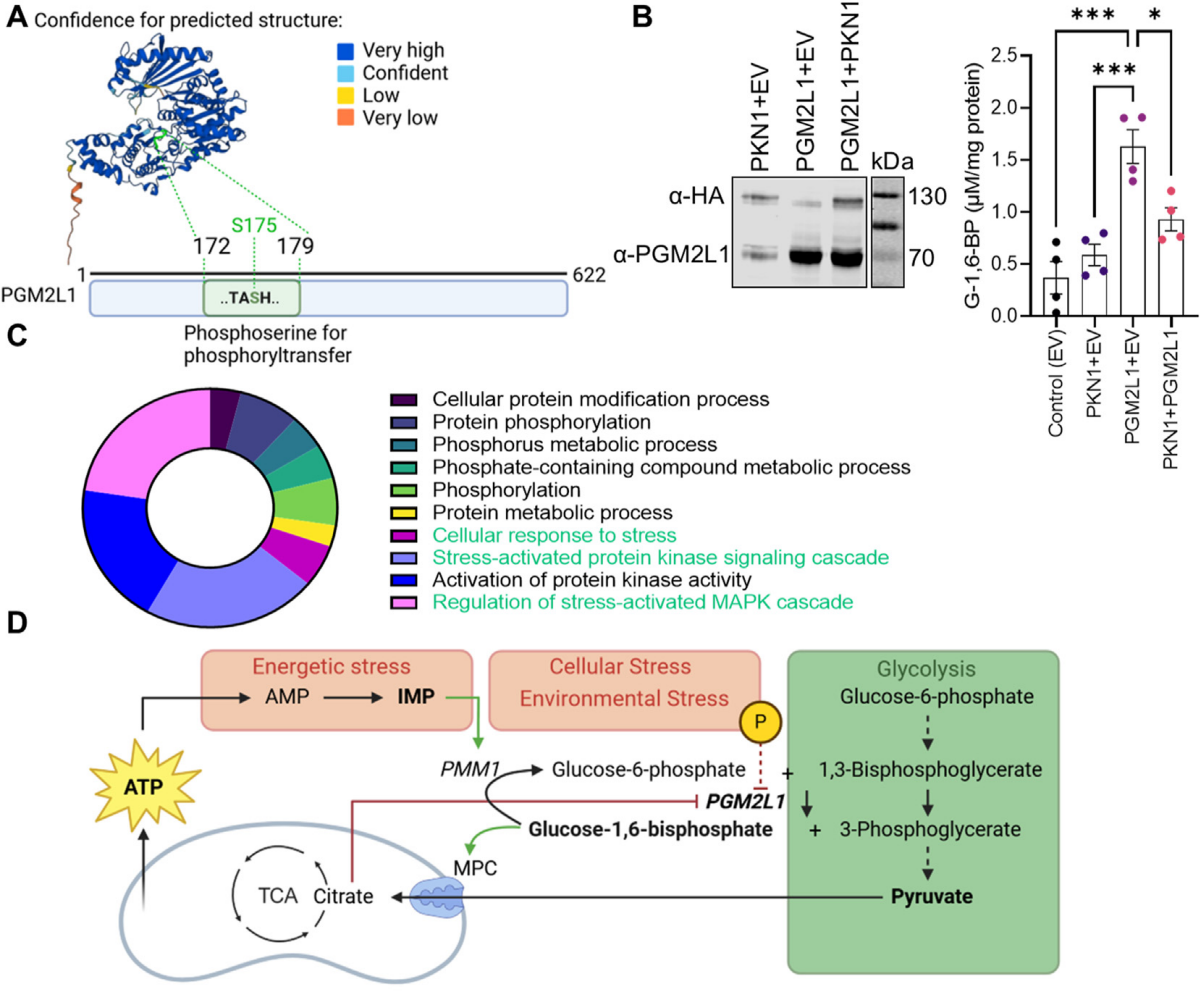
**Regulation of PGM2L1 activity.** (**A**) Human PGM2L1 protein structure was predicted with AlphaFold Monomer v2.0 pipeline. The amino acids T173-H176 are shown in green in the predicted structure and encompass the predicted phosphorylation site S175. (**B**) Human SH-SY5Y cells were transfected with empty vectors (EV for the PKN1 and PGM2L1 plasmids; Control), HA-tagged PKN1+EV (for PGM2L1 plasmid), untagged PGM2L1+EV (for PKN1 plasmid) and PKN1+PGM2L1 and G-1,6-BP levels were analyzed (one way ANOVA with Holm-Šídák's multiple comparisons test ((∗) P < 0.05, (∗∗∗) P < 0.001). Blot is representative of 3 separate experiments. (**C**) The top 50 (rank) kinases predicted to phosphorylate human PGM2L1 S175 were analyzed with WebGestalt, using Network-Topology based analysis and PPI Biogrid. A pie chart summarizing the enrichment ratio (% of total) of the top 10 predicted networks are shown. (**D**) Scheme summarizing the metabolic processes involved in PGM2L1 regulation. Glycolysis, shown in green, has a stimulatory- and metabolic stress an inhibitory effect on G-1,6-BP levels, due to degradation by PMM1. Additionally PGM2L1 is inhibited by citrate, encompassing a potential feedback inhibition. The phosphorylation of S175 is further predicted to inhibit PGM2L1 activity. All data are presented as scatter blot with mean ± S.E.M. A and D were prepared in BioRender.

Up to date not much is known about the regulation of PGM2L1 in the brain. Human PGM2L1 protein structure was predicted with AlphaFold Monomer v2.0 pipeline [42] and a scheme of the PGM2L1 amino acids with the active site involved in the transfer of the phosphate group from 1,3-bisphosphoglycerate to a hexose-1 or hexose-6-phosphate [5,43] is shown (Figure 4A). The “TASHNP” motif is common to members of the α-d-phosphohexomutase family and is important for the reaction mechanism, that involves a transient phosphorylation of the serine [5,44]. The fact that PGM2L1 also comprises a serine in a similar motif (TASHNR, S175 is shown in green in Figure 4A) as found in phosphoglucomutase 1-related enzymes [43] aligns with previous findings that PGM2L1 is phosphorylated on a serine residue by the transfer of the 1-phospho group of 1,3-bisphosphoglycerate [45]. In that respect it is interesting that the S175 of human PGM2L1 can be phosphorylated by kinases [46,47]. A predicated upstream kinase is protein kinase N1 (PKN1) [46] and indeed PKN-mediated phosphorylation of PGM2L1 S175 has been confirmed in a kinase interacting substrate screen [48]. To test if PKN1 regulates PGM2L1 activity we overexpressed both proteins in human SH-SY5Y cells. We found that, while PGM2L1 significantly upregulated cellular G-1,6-BP levels, co-expression with PKN1 reduced this effect (Figure 4B), showing that PKN1 exerts an inhibitory effect on PGM2L1 activity.

We further used the top 50 ranked protein kinases predicted to phosphorylate human PGM2L1 S175 [46] (Supplemental Table 2) for a network-topology based analysis employing WebGestalt [49] (Figure 4C). Interestingly, the predicted upstream kinases generally belong to networks involved in protein phosphorylation and metabolic processes as well as networks involved in cellular stress (Figure 4C).

Therefore G-1,6-BP is ideally positioned to integrate information about (1) the glycolytic flux, which positively regulates G-1,6-BP levels, (2) metabolic stress with relation to IMP production and (3) extracellular signals via phosphorylation on S175 (Figure 4D). Additionally, it has been shown that citrate, isocitrate and cis-aconitate inhibit PGM2L1 activity by competition with 1,3-bisphosphoglycerate [50] potentially providing a feedback inhibition. This makes G-1,6-BP an ideal candidate for signaling to the mitochondria about the bioavailability of pyruvate and the cellular energetic state (Figure 4D).

## 4 Discussion

Pyruvate occupies a central metabolic node due to its position at the crossroads of glycolysis and the TCA cycle and the precise regulation of mitochondrial pyruvate uptake is fundamental for cellular energy metabolism [51]. Here we uncovered a novel mechanism involving G-1,6-BP, a metabolite that is enriched in the brain and sensitive to the glycolytic flux as well as the cellular energy state [9]. We show that G-1,6-BP, at physiological concentrations found in the brain, has a stimulatory effect on mitochondrial pyruvate uptake and pyruvate-induced mitochondrial respiration. We further provide evidence that PGM2L1 is important for post-ischemic neuronal survival in an *in vitro* stroke model and that PMG2L1 activity can be fine-tuned by protein kinases, specifically PKN1.

Mitochondrial pyruvate transport through MPC is sensitive to cytosolic pyruvate concentrations [52] and inhibited by acetylation [53] as well as branched chain ketoacids [27]. Therefore, MPC regulation by metabolites might serve as a general mechanism to sensitize mitochondria to the bioavailability of certain substrates. Unlike branched chain ketoacids, which interact with MPC1 [27], G-1,6-BP can bind to both MPC1 and MPC2, albeit with a higher affinity for MPC2. While the exact mechanism of G-1,6-BP-mediated augmentation of mitochondrial pyruvate uptake remains to be established, it might involve the stabilization of MPC1-MPC2 heterodimers. In that respect it should be noted, that PGM2L1 can also synthesize mannose-1,6-bisphosphate and ribose-1,5-bisphosphate [5], both of which might also serve as metabolic regulators. Indeed, ribose-1,5-bisphosphate has been shown to regulate phosphofructokinase activity [54]. While our studies involving PGM2L1 overexpression or knockdown cannot fully exclude an additional effect of other hexose or pentose bisphosphates on mitochondrial pyruvate uptake, we were able to show the specific effect of G-1,6-BP on MPC activity as well as mitochondrial pyruvate uptake and respiration by exogenous addition of this metabolite to permeabilized cells and isolated mitochondria.

Another important detail is that the G-1,6-BP phosphatase PMM1 is sensitive to IMP but not to adenosine monophosphate [9]. This is especially interesting since stimulation of neuronal activity specifically results in elevation of IMP levels [55]. Thus G-1,6-BP might sense the energetic state of the cell also during physiological stress, such as electrophysiological activity. In that context, some of the upstream kinases for PGM2L1 S175 are known to be involved in synaptic functions, such as TRAF2 and NCK interacting kinase [56], PKN1 [57] or protein kinase C iota [58]. Furthermore, another potential phosphorylation site T173 [46,47], which is also part of the TASHNR motif, is predicted to be targeted by calcium/calmodulin-stimulated protein kinase kinase I and II, two well-established players in neuronal transmission [59]. Therefore, future studies should be directed to analyze if G-1,6-BP and other hexose- or pentose-bisphosphate levels change during electrical activity and if it might regulate mitochondrial uptake of pyruvate and other TCA substrates such as glutamine/glutamate or fatty acids.

Notably we could also detect PGM2L1 in the cytosol. In addition human erythrocytes have measurable G-1,6-BP levels [60]. While G-1,6-BP in erythrocytes might not be a free metabolite, but instead bound to hemoglobin like other phosphorylated sugars [9], it still suggests that G-1,6-BP is involved in processes other than mitochondrial pyruvate uptake.

In summary our data reveal a novel mechanism by which neurons can couple glycolysis-derived pyruvate to the TCA cycle. G-1,6-BP is ideally positioned to integrate information about the glycolytic flux, the cell's energetic state, and upstream signaling cascades, thereby offering many regulatory means to fine-tune this critical metabolic step.

## Funding

This research was funded by the Austrian Science Fund (FWF), grant numbers T1091 (Grant-DOI: 10.55776/T1091) and P31085-B26 (Grant-DOI 10.55776/P31085) and ERC ADG #786462 – HOPE. M.K. thanks the Tyrolian Research Fund (Project No. 18903) for financial support.

## CRediT authorship contribution statement

**Motahareh Solina Safari:** Investigation, Formal analysis. **Priska Woerl:** Investigation, Formal analysis. **Carolin Garmsiri:** Investigation, Formal analysis. **Dido Weber:** Investigation, Formal analysis. **Marcel Kwiatkowski:** Resources, Methodology, Investigation, Funding acquisition, Formal analysis. **Madlen Hotze:** Methodology, Investigation, Formal analysis. **Louisa Kuenkel:** Investigation. **Luisa Lang:** Investigation, Formal analysis. **Matthias Erlacher:** Methodology. **Ellen Gelpi:** Resources, Methodology. **Johannes A. Hainfellner:** Resources. **Gottfried Baier:** Supervision, Resources, Methodology, Funding acquisition. **Gabriele Baier-Bitterlich:** Writing – review & editing, Writing – original draft, Supervision, Project administration, Funding acquisition, Conceptualization. **Stephanie zur Nedden:** Writing – review & editing, Writing – original draft, Validation, Supervision, Project administration, Methodology, Investigation, Funding acquisition, Formal analysis, Conceptualization.

## Declaration of competing interest

The authors declare that they have no known competing financial interests or personal relationships that could have appeared to influence the work reported in this paper.

## Data Availability

Data will be made available on request.
