# Supplementary material for: Glucose-1,6-bisphosphate: A new gatekeeper of cerebral mitochondrial pyruvate uptake
Source: Mol Metab. 2024 Aug 24;88:102018. doi: 10.1016/j.molmet.2024.102018 (PMC11404074; doi:10.1016/j.molmet.2024.102018)
Supplement: Multimedia component 3 [file mmc3.pdf]

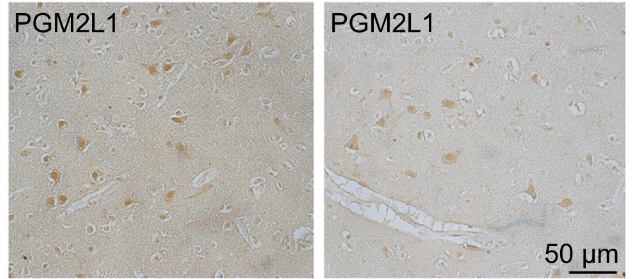

Supplemental Figure 1: Staining of human frontal cortex with PGM2L1 (N=2).

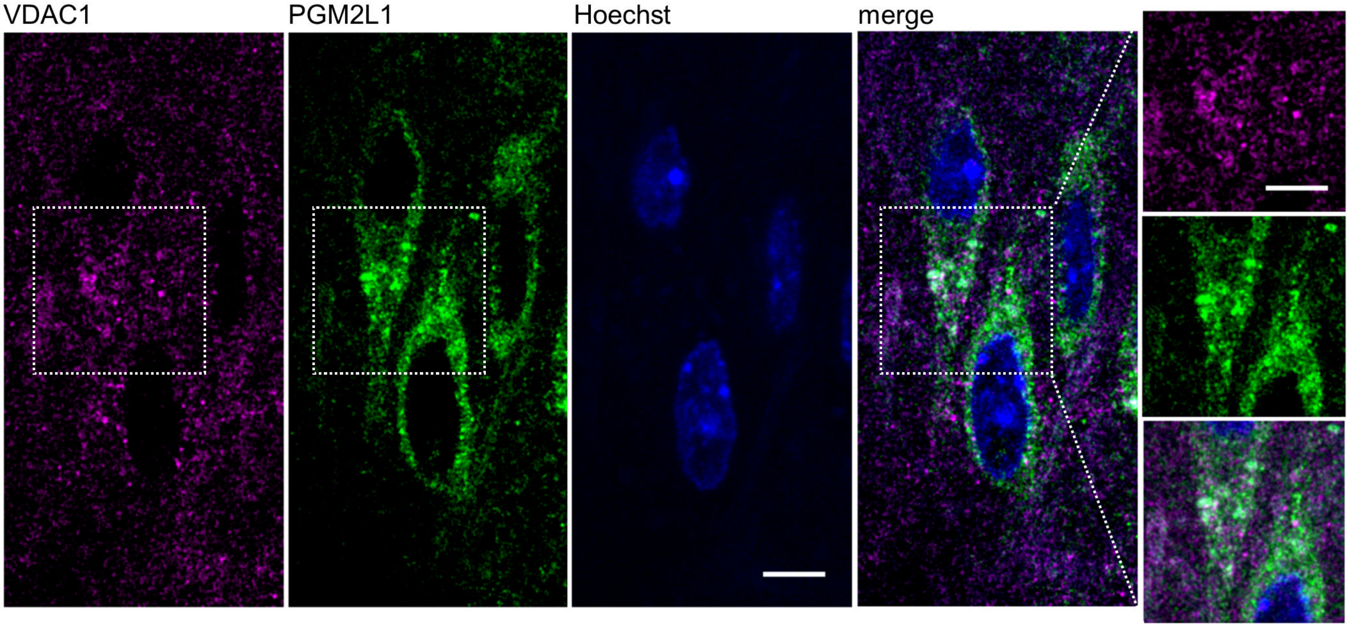

Supplemental Figure 2: Partial colocalization of PGM2L1 and the mitochondrial marker VDAC1 was confirmed in mouse brain. Brains were fixed in PFA overnight, incubated in sucrose (30%) and embedded in O.C.T.. Sections were cut at 20 µm thickness and transferred on polysin slides (Thermo Fisher). Staining was performed as described in section 2.6. Scale bar refers to 5 µm. Image is representative of 2 separate experiments.

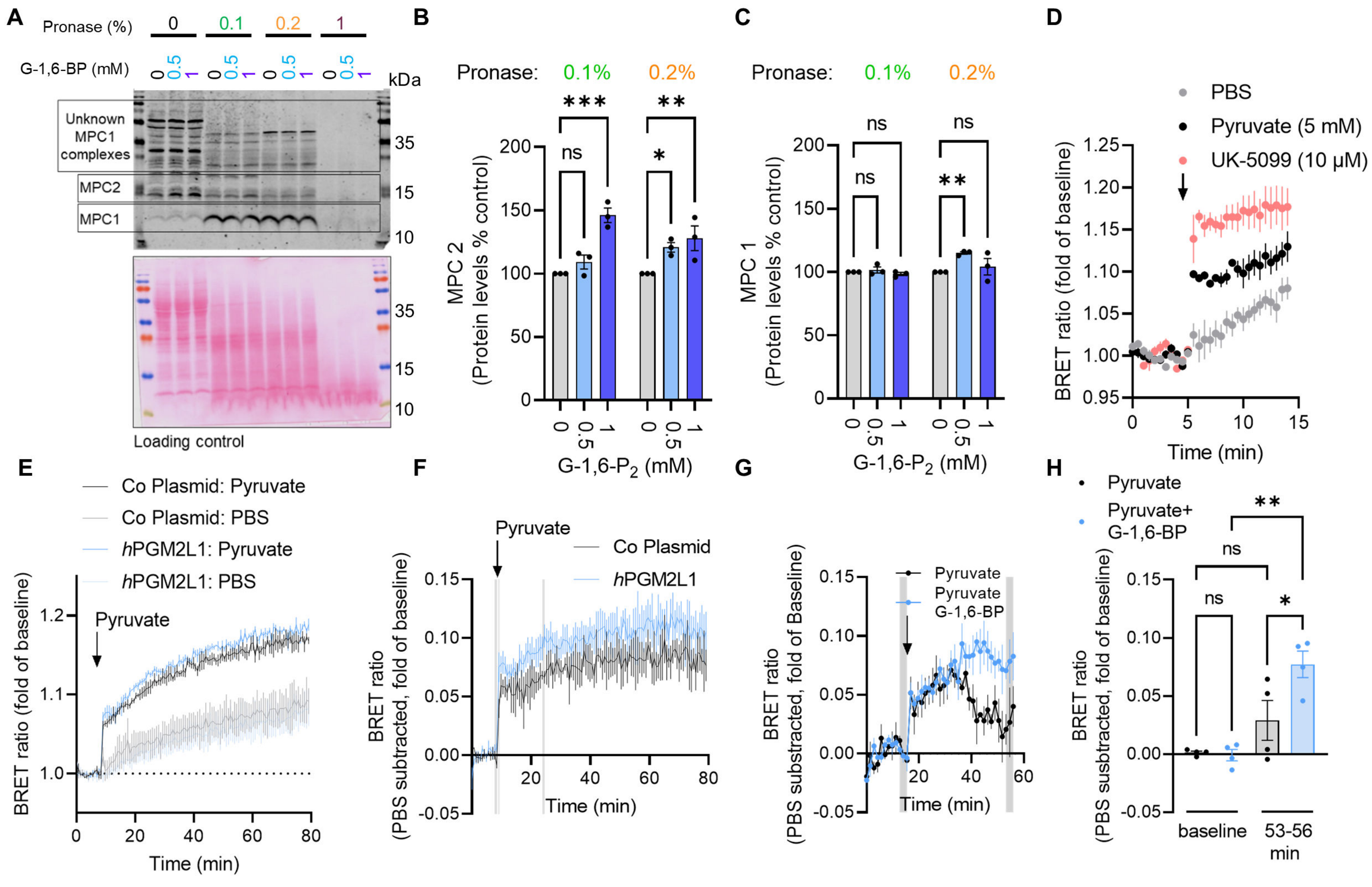

Supplemental Figure 3: (**A**) A DARTs assay was performed with hippocampal extracts and addition of exogenous Pronase at 0-1% Pronase/mg protein. Since loading controls (actin, GAPDH and tubulin) were degraded at low pronase (0.1%) concentrations, we used ponceau red staining as a loading control. (**B**) MPC2 was significantly protected by G-1,6-P2 from Pronase-induced degradation (two way ANOVA, treatment (G-1,6-P2) P<0.0001, (pronase) P>0.05, (interaction) P<0.05, Holm-Šídák's multiple comparisons test: ((*) P< 0.05, (**) P< 0.01, (***) P<0.001, (ns) not significant).). (**C**) MPC1 was moderately protected by G-1,6-P2 from Pronase (0.2%)-induced degradation (two way ANOVA, (G-1,6-P2) P<0.05, (pronase) P<0.05, (interaction) P>0.05, Holm-Šídák's multiple comparisons test: (**) P<0.01, (ns) not significant). (**D**) HEK cells, stably expressing the RESPYR constructs MPC1-venus (MPC1V) and MPC2-RLuc8 (MPC2R), were tested with pyruvate (5 mM) and UK-5099 (10 µM), both of which increased the BRET ratio (N=3). (**E**) The response of HEK MPC1V/MPC2R cells transfected with co Plasmid or *h*PGM2L1 were compared after addition (see arrowhead) of pyruvate (5 mM) or PBS. Values are expressed as fold of baseline. N=3. (**F**) The values in (E) for PBS were subtracted from the pyruvate-treated wells. (**G**) HEK MPC1V/MPC2R were permeabilized with saponin (25 µg/ml) for 15 min and pyruvate (5 mM) pyruvate with G-1,6-BP (100 µM) was added. The BRET ratio is expressed as fold of baseline with the respective PBS responses subtracted. The delayed effect of G-1,6-BP might be due to diffusion restrictions to or into mitochondria. (**H**) The mean of the 3 baseline responses and 3 responses at 53-56 min were compared (areas marked in grey in (G), one way ANOVA with Holm-Šídák's multiple comparisons test: (*) P< 0.05, (**) P< 0.01, (ns) not significant). All data is represented as mean ± S.E.M..
